# China’s Land Uses in the Multi-Region Input–Output Framework

**DOI:** 10.3390/ijerph16162940

**Published:** 2019-08-16

**Authors:** Chao Bao, Mutian Xu, Siao Sun

**Affiliations:** 1 Key Laboratory of Regional Sustainable Development Modeling, Institute of Geographic Sciences and Natural Resource Research, Chinese Academy of Sciences, Beijing 100101, China; 2 College of Resources and Environment, University of Chinese Academy of Sciences, Beijing 100049, China

**Keywords:** multi-type land uses, inter-provincial trades, land transfers, input–output analysis, footprint, virtual land

## Abstract

The finite resource of land is subject to competing pressures from food demand, urbanization, and ecosystem service provision. Linking the land resource use to the whole production chain and final consumption of various products and services offers a new perspective to understand and manage land uses. This study conducted a systematic analysis of land uses at the provincial level in China using the multi-region input–output model in 2012. Land use patterns related to the sectoral production and consumption in different provinces were examined. The results indicated that the land use transfers between different provinces in China have formed a highly interacting network. Products and services involved in the inter-provincial trades in China contained 2.3 million km^2^ land uses, which constituted approximately 40% of the total national land uses that were finally consumed in China. Agriculture was the most direct land use intensive sector, and industry was the most indirect land use intensive sector. Land resource-scarce provinces with low per capita land availability have outsourced parts of their land uses by net importing lands from other provinces. The results have important policy implications towards sustainable land uses in China.

## 1. Introduction

Land is one of the most critical resources in economic development and environmental ecosystem provision [1]. The land, which is a finite resource, is subject to competing pressures from increasing food demand, urbanization, and provision of ecosystem services, along with the growing population and developing economy [2,3]. This is a worldwide problem. Particularly in China, with the largest population and booming economy, the pressure on meeting the ever-increasing land demands is increasing [4]. Given uneven spatial distributions of population, croplands, and industries in China, the challenge of reducing the risks of land resource shortage also presents high geographical differences [5]. Therefore, sustainable land management and development is high on the agenda of the state and local governments in China (such as the thirteenth five-year plans at the national and local levels) committed to green development for a resource-conserving and environmental-friendly society [6].

With the advancement of transportation and information advancement, processes of production and consumption are increasingly fragmented, and the supply chains transcend political and geographical borders, benefiting from competitive advantages in many regions [7]. As the economic links between regions continue to intensify through trades of various goods, the transboundary dependencies of production and consumption have become an increasingly important driver of environmental impacts, including land resource uses [8]. Linking the land resource use to the whole production chain and final consumption of various products and services offers a new perspective to understand and manage the environmental impact of anthropogenic activities [9,10,11]. Similar to water, energy, air pollution, and other issues [12,13,14,15], the accounting of land resources embedded into commodities in trades helps identify the displacement of land uses and appropriate responses of the resource consumption based on final consumption of inhabitants, and is; thus, useful in informing policymakers on the magnitude of external environmental burdens imposed by certain regions [16].

For accounting resource or material flows embedded into products and services in the trade, the input–output analysis (IOA) method, which is able to assign resource or material flows to final consumers based on the diverse linkages among industries in the production chains [17], has been commonly applied. Using this approach, sectoral direct and indirect flows can be quantified and traced along an interconnected economic network [18,19]. In the context of multiple regions, the multi-regional input–output (MRIO) method has been developed based on the IOA, which tracks the direct and indirect flows of resources or other environmental impacts between sectors and regions [20]. A more direct approach calculates embodied land flows by multiplying the land use per unit of products and the amount of traded products. In comparison to this direct approach that often focuses on croplands or agricultural lands, the IOA- or MRIO-based methods include the developed land for industrial and tertiary uses, which is an overlooked but increasingly important land use category [21].

The IOA or MRIO approaches have been applied to land use analysis at the international, national and intra-national levels [22,23,24,25,26], providing land resource trading information as well as the dependence and supporting relationships of land resources between different regions. In particular for China, the impact of domestic consumption and international trade on agricultural land uses has been analyzed based on China’s input–output table or the global MRIO table [19,22,27,28]. At the provincial level, a few recent studies have measured the lands hidden in traded crops and examined the pattern and characteristics of inter-provincial land trade flows based on the economic trade network and land intensity data [5,29]. These studies focused mainly on agricultural land uses, but ignored other types of land uses. A comprehensive analysis of land trade flows at the provincial level in China, including industrial and tertiary land uses in the full production chains, is not yet implemented.

In principle, the trade between regions allows for land resource transfers across regional borders, providing opportunities for mitigating resource shortages in resource scarce regions by importing the resource from other regions [2,28]. However, because the trade between regions is controlled by comparative advantages determined from a combination of many natural and socio-economic factors (e.g., natural resource abundance, capital and technology), land resource is not necessarily transferred downward the gradient of land resource abundance. A few previous studies at the global scale concluded that the affluence is the main driver for the global displacement of land uses [24,30]. However, the pattern and influencing factors associated with the inter-provincial land use transfers in China remain unknown.

In order to bridge the above gaps, this study analyzed China’s land uses based on the latest China MRIO table in 2012, which is essential for understanding the current land use patterns by linking the land resource use to the whole production chain and final consumption. The main contributions of this study lied in: (1) Inter-provincial land resource trades of multiple land uses were assessed and the trade network was constructed. (2) Influencing factors that were associated to the net land use trades in China were examined to help illustrate how the land use transfer network was shaped. The results of this first comprehensive land use trade study in China have important policy implications towards sustainable land management.

## 2. Materials and Methods

### 2.1. Land Use Accounting in a Multi-Region Input–Output Framework

The environmental extended input–output model was used to calculate land uses embodied in trades and footprints of land uses in individual provinces in China. In the MRIO table with *R* regions and *N* economic sectors, the sectoral outputs in each region are used as intermediate inputs and for final consumption in all the provinces:

(1) $$\left[\begin{array}{c} x^{1}\\ \vdots \\ x^{R} \end{array}\right]=\left[\begin{array}{ccc} A^{11} & \cdots & A^{1R}\\ \vdots & \ddots & \vdots \\ A^{R1} & \cdots & A^{RR} \end{array}\right]\left[\begin{array}{c} x^{1}\\ \vdots \\ x^{R} \end{array}\right]+\left[\begin{array}{c} y^{11}+\underset{s\neq 1}{\sum }y^{1s}+ex^{1}\\ \vdots \\ y^{RR}+\underset{s\neq R}{\sum }y^{Rs}+ex^{R} \end{array}\right],$$

where *x^R^* is an *N*×1 vector representing the output of *N* sectors in province *r*, *A^Rs^* is an *N* × *N* matrix of technical coefficients, with the element $a_{ij}^{Rs}$ representing the amount of input from sector *i* in province *R* to produce one monetary unit of output in sector *j* in province *s*, *y^RR^* is an *N* × 1 vector indicating domestic final consumption in province *r*, *y^Rs^* is the products from province *r* to province *s* for final consumption, and *ex^R^* is an *N* × 1 vector representing products exported abroad. Equation (1) can be solved as:

(2) $$X={\left(I-A\right)}^{-1}Y,$$

where *X* = [*x*^1^, *x*^2^, …, *x^R^*]^T^ in Equation (1), *I* is the identity matrix, and ${\left(I-A\right)}^{-1}$ is the Leontief inverse matrix, $Y={\left[\underset{s}{\sum }y^{1s}+ex^{1},\;\underset{s}{\sum }y^{2s}+ex^{2},\;\ldots ,\underset{s}{\sum }y^{Rs}+ex^{R}\right]}^{T}$.

Land uses *LU* are introduced in Equation (2) and the equation can be written as:

(3) $$LU=DX=D{\left(I-A\right)}^{-1}Y=TY,$$

where *LU* = [*lu*^1^, *lu*^2^, …, *lu^R^*]^T^, *lu^r^* is an *N* × 1 vector representing the total land uses in the *N* sectors in province *r*. *D* is a diagonal matrix with the diagonal elements [*d*^1^, *d*^2^, …, *d*^N^] denoting the direct land use intensity, with the values *d^r^* representing the direct land use per unit of output in the *N* sectors in province *r*, *T* is a matrix denoting the total land use intensities (including both indirect and direct uses), where the element *t^rs^* is the land required from province *r* to produce one unit of a product in province *s* for final consumption.

The land use from province *r* to province *s* $lt^{rs}$ embodied in commodities by trade can be computed as:

(4) $$lt^{rs}=\underset{i}{\sum }t^{ri}y^{is},$$

where $t^{ri}$ is a row vector, and $y^{is}$ is a column vector. Similarly, the land use embodied in commodities from province *r* to foreign countries is:

(5) $$lex^{r}=\underset{i}{\sum }t^{ri}ex^{i}.$$

The consumption-based land use of province *r* (denoted by $luf^{r}$) can be calculated as the sum of the local land use and net land use transfers due to trade:

(6) $$luf^{r}=lu^{r}+\underset{s\neq r}{\sum }lt^{sr}-\underset{s\neq r}{\sum }lt^{rs}-lex^{r}.$$

Therefore, based on the Leontief inverse matrix and direct land uses in provinces, the environmental extended input–output model enables the appropriations of the direct land uses in provinces to final consumers of different types of land uses.

### 2.2. Bivariate Map of Net Land Use Transfers and Possible Influencing Factors

Provinces import and export land uses embodied in commodity trades. The net land use transfer (i.e., the difference of land use export and import) indicates whether a province relies on external resource uses for final consumption or provides other provinces with land use export. Analysis of relations between the net land use transfers and possible influencing factors helps understand how the land use transfer network is formed, and thus is a prerequisite for policy implications. A number of variables were selected as possible influencing factors to study their relationships with net land use transfers based on data availability and the relevance. Variables were selected at two dimensions to characterize: (1) Natural endowment and (2) socioeconomic conditions in different provinces. Variables characterizing natural endowment included land use per capita and annual precipitation. Variables for socioeconomic conditions included economic density (the ratio of GDP to area size), population density (the ratio of population to areas size) and land use intensity (the ratio of land use to GDP) [31,32]. The bivariate map, which displays the net land use trade and one possible influencing factor on a single map, was used to graphically illustrate the relationship between two spatially distributed variables. The rank correlation coefficient, which is a nonparametric measure of the correlation, was calculated between the net land use transfer and one possible influencing factor, and the statistical significance of the correlation of the two variables was tested.

### 2.3. Data Sources

The main data sources used in this study were the multi-region input–output (MRIO) table [33] and provincial-level sectoral land use data in China. The latest China MRIO table in 2012 provided monetary transactions of goods and services among 8 sectors in 31 provinces in mainland China (regions of Hong Kong, Macao, and Taiwan are not included due to data unavailability). The 8 economic sectors can be grouped into 3 categories: agricultural (one sector comprising farming, forestry, animal husbandry, and fishery), industrial (two sectors including industry and construction), and tertiary (comprising 5 sub-sectors including wholesale and retail, transportation and storage, accommodation and catering, information technology and software, and other services) categories.

Land uses data corresponding to the sectors in the MRIO table in different provinces were inferred from multiple data sources. Data of agricultural lands (mainly including crop land, grassland, woods, and garden plot) referred to agricultural land use data in 2012 from China Statistical Yearbook [34]. Lumped industrial and tertiary land uses were obtained from Urban and Rural Construction Statistical Yearbook 2013 [35] by aggregating corresponding sectoral land uses in cities, counties, and rural areas. Industrial land use is mainly the land use for industrial manufacturing, whereas tertiary land use includes land uses for administration and public services, logistics and warehouse, road, street and transportation, and municipal utilities. The lumped industrial and tertiary land uses in different provinces were then disaggregated into land uses in the sub-sectors proportional to the total outputs of corresponding sub-sectors based on the MRIO table. Because land uses in the 3 aggregated agricultural, industrial, and tertiary sectors can generally reveal the main properties, results for land uses were often reported for these 3 aggregated sectors, to facilitate the discussion.

The summarized statistics and data sources of the selected possible influencing factors of net land use transfers were listed in Table 1.

## 3. Results and Discussion

### 3.1. Direct and Total Land Use Intensities

Direct land use intensity refers to the amount of direct local land uses to produce one monetary unit of products (the variable *D* in Equation (3)). Total land use intensity is defined as the amount of the total land input per monetary unit of products for final consumption, including both direct and indirect land uses through the whole production chain (the variable *T* in Equation (4)). The difference between the total and direct land use intensities represents indirect land use intensity, which is the land use for intermediate input production that is required for producing one monetary unit of outputs for final consumption. Table 2 listed the direct and total land use intensities for agricultural, industrial, and tertiary products in different provinces in China.

The means of the direct land use intensities of agricultural, industrial, and tertiary products in different provinces were 3496.7, 0.1, and 0.3 km^2^/billion yuan, respectively. Agriculture was the most direct land use intensive sector, with the direct land use intensity four orders of magnitude higher than those of industrial and tertiary sectors. Land uses for industrial and tertiary sectors presented higher efficiency in comparison to agricultural land uses. The reasons were probably twofold: (1) The industrial and tertiary products are in nature less land-consuming; (2) productions for industrial and tertiary products often occur in urban areas, where lands are usually limited and intensively arranged. The total land use intensities were commonly higher than corresponding direct land use intensities. The means of the total land use intensities of agricultural, industrial, and tertiary sectors in different provinces were 4228.4, 11,210.8, and 77.6 km^2^/billion yuan, respectively. Industry was the most total land use intensive sector. The total land use intensity for industrial products was several orders of magnitude higher than its direct land use intensity. This can be explained by the fact that large amounts of agricultural products containing intensive land uses were used as intermediate input in industrial production chains. The total land use intensity of the tertiary products was the lowest. The average ratios of indirect land uses to direct land uses for agricultural, industrial, and tertiary sectors were 0.3, 96,484.7, and 197.8, respectively. While the indirect land use for agriculture was slightly lower than its direct land use, indirect land uses in industrial and tertiary sectors constituted a dominating proportion of the total land uses, implying the importance of including indirect land uses in the production processes for appropriating land uses of different products. Neglecting indict land uses will greatly underestimate land use requirement for commodity production, particularly for industrial and tertiary products.

Direct and total land use intensities in different provinces presented high heterogeneity. The relative standard deviations of direct land use intensities in different provinces for the agricultural, industrial and tertiary sectors were 3.69, 0.34, and 0.33, respectively. The variability of provincial direct land use intensity for the agricultural sector was the largest, likely due to distinct climatic and geographic conditions, varying cultivated crops, agricultural technology gaps and different agricultural management modes. The relative standard deviations of the total land use intensities for the agricultural, industrial, and tertiary sectors were 3.74, 1.60, and 2.82, respectively, which were higher than those of the direct land use intensities for corresponding sectors. The indirect land uses included in the total land uses led to wider gaps of provincial land use intensities, particularly for the industrial and tertiary sectors. High land use intensities often occurred in less developed provinces. Tibet, Qinghai, and Xinjiang were the provinces with the highest total agricultural, industrial, and tertiary land use intensities. By contrast, low land use intensities were found in more developed provinces. Provinces with the lowest total agricultural land use intensities were Jiangsu, Shanghai, and Tianjin. Guangdong, Shanghai, and Tianjin were the top three provinces with the lowest total industrial land use intensities. Jiangsu, Shanghai, and Beijing had the lowest total tertiary land use intensities.

### 3.2. Production and Consumption-Based Land Uses

**Figure 1:**
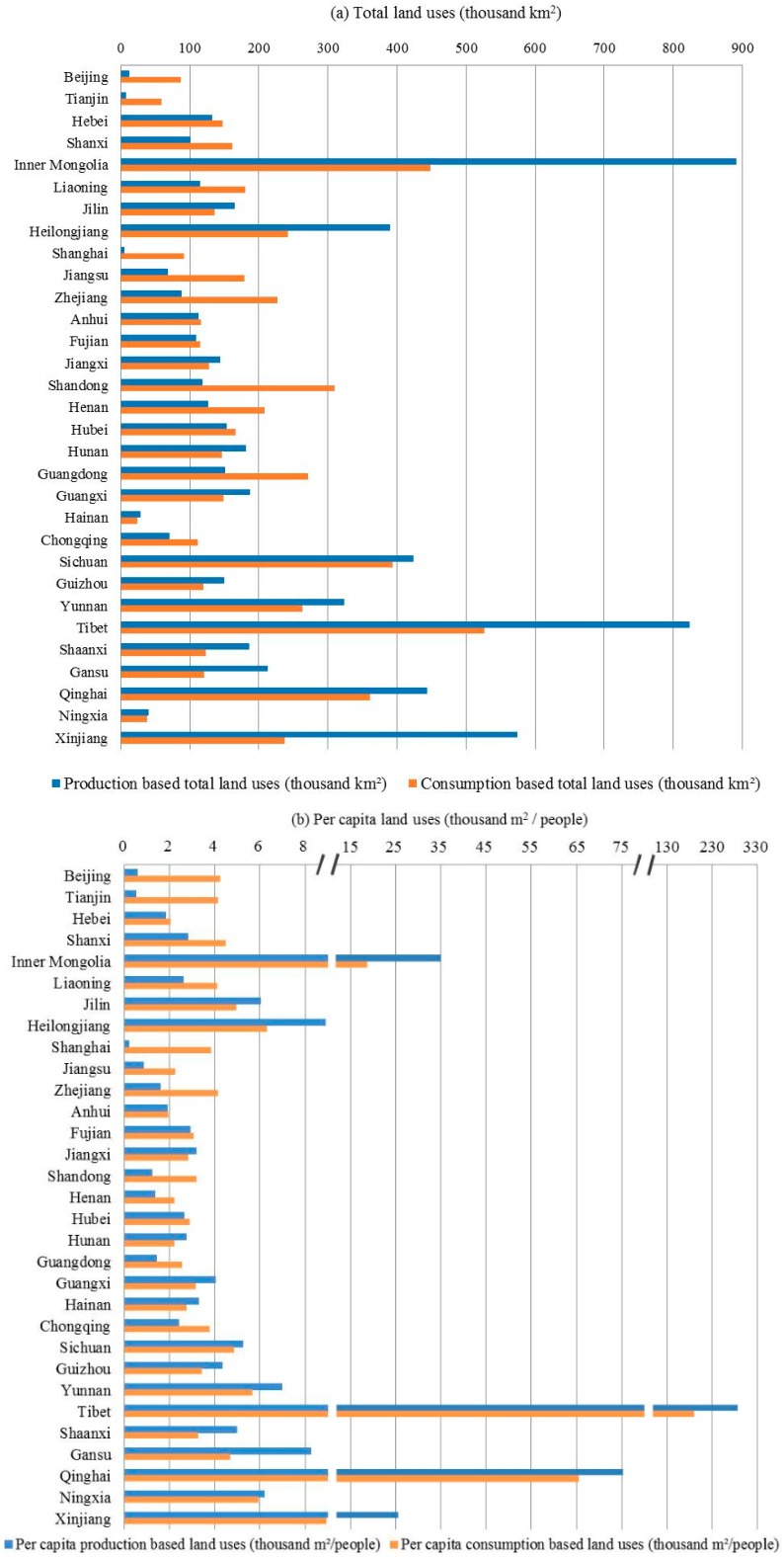
Production- and consumption-based land uses in provinces in China: (**a**) Total land uses; (**b**) per capita land uses.

There are two alternative ways to quantify land uses (i.e., from the production and consumption perspectives). In a geographically-delineated region (e.g., a country or a province), the production-based land use is the domestic land use for sectoral production activities that takes place in that area (the variable *lu* in Equation (3)), whereas the consumption-based land use refers to the land resources embodied in products and services that are finally consumed by local residents [21,30], including both production-based internal and external land uses (*luf* in Equation (6)). While the production-based land use is a more direct measure for local land use pressures, the consumption-based land use appropriates the responsibility of land use pressures to the final consumers [25,36]. Under the framework of the consumption-based land use, a region’s land uses may finally be linked to the consumption of products in other regions. Analysis of both production- and consumption-based land uses will contribute to a better understanding of connections between producing and consuming regions mediated by trades [37]. The total land uses and per capita land uses based on the production and consumption perspectives were presented in Figure 1.

In Figure 1a for the total land uses in different provinces in China, the production-based land uses distributed in the range between 4.9 and 894.1 thousand km^2^, and the consumption-based land uses ranged between 24.5 and 526.8 thousand km^2^, noting that land uses embodied in product imports via international trades were not accounted in this study. The gap between the production- and consumption-based land uses in one province represented the trade imbalance of land uses embodied in commodities [25]. Sixteen provinces had production-based land uses larger than consumption-based land uses, among which, 14 provinces belonged to the less developed regions with the per capita GDP lower than the national average. The other two provinces were Inner Mongolia and Jilin. Provinces with higher consumption-based land uses than production-based land uses were mostly more developed provinces with the per capita GDP higher than the national average [34]. This phenomenon was consistent with “land grabbing”, referring to the land displacement from poor regions to wealthy regions [38].

In Figure 1b for per capita land uses at the provincial level, the production-based per capita land uses distributed between 0.2 and 267.6 thousand m^2^, and the consumption-based per capita land uses were between 1.9 and 171.0 thousand m^2^. The variability in per capita consumption-based land uses was smaller than that in per capita production-based land uses, suggesting that inter-provincial trades led to land use displacement tending to reduce the inequality of per capita land uses between provinces, given the current population distribution in different provinces. Per capita production- and consumption-based land uses in Tibet were the largest, followed by Qinghai, Inner Mongolia, and Xinjiang, which were exclusively located in western China, with relatively abundant land resources and few inhabitants. Per capita production-based land use in Shanghai was the smallest. Other provinces with per capita production-based land uses below 1.0 thousand m^2^ included Tianjin, Beijing, and Jiangsu. These were the most affluent provinces in China with intensive industries and dense populations. By importing net land uses through inter-provincial trades, per capita consumption-based land uses in these affluent provinces were increased by 3~19 times in comparison to their per capita production-based land uses. Per capita consumption-based land uses in these provinces were between 3.8 and 6.3 thousand m^2^, which were above the national average level.

**Figure 2:**
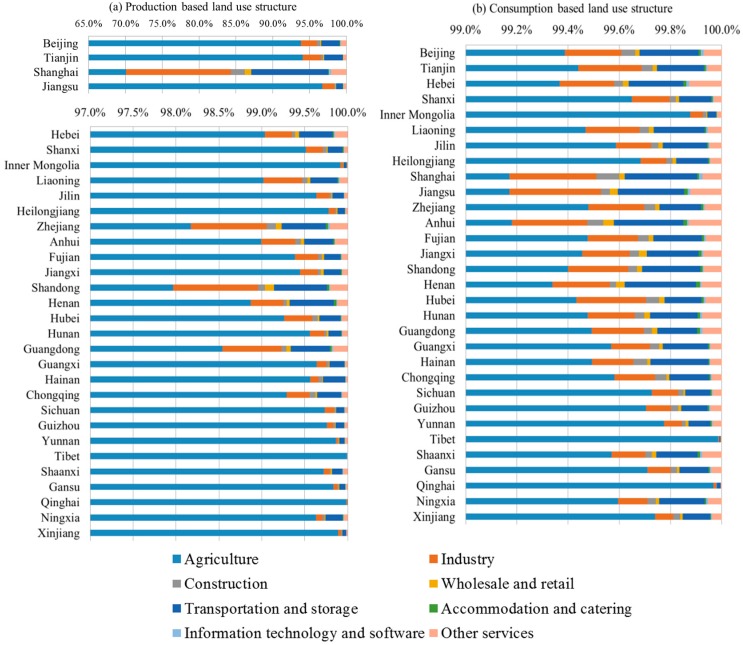
The land use structures in provinces in China: (**a**) Production-based land uses; (**b**) consumption-based land uses.

The land use structures of different proivnces were shown in Figure 2. Land uses were dominated by agriculture. In Figure 2a for the production-based land uses, agricultural land uses consitututed between 70.11% and 99.99% of the total land uses. In Figure 2b for the consumption-based land uses, the shares of agricultural land uses were between 99.17% and 99.99%. Shanghai had the smallest shares of agricutual land uses from both production and consumption perspecptives, whereas the largest proportions of agricutltural land uses occurred in Tibet. The seven other non-agricultural sectors represented a minor proportion of land uses, in which industry and transportation and storage were ranked top land users. The shares of production- and consumption-based sectoral land uses in different provinces were highly correlated, which can be explained by the fact that a significant share of products and services were finally consumed locally. According to the land use accounting at the provincial level, between 25.6% and 86.3% of the total land uses embodied in products and services were consumed by local inhabitants in different provinces. In 24 provinces, more than a half of the land uses embodied in products and services were finally consumed by local inhabitants.

### 3.3. Land Use Transfers in Inter-Provincial Trades

**Figure 3:**
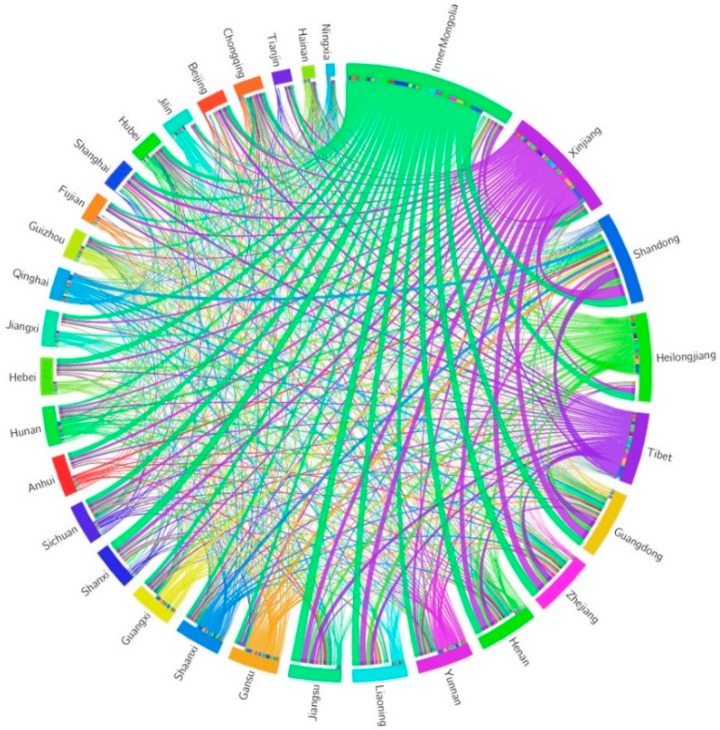
Inter-provincial land use transfers in China.

The land uses that are embodied in products and services in the domestic production chains are finally consumed by local inhabitants or transferred to other provinces and countries by inter-provincial and international trades. The land uses that were finally consumed in China was about 5.9 million km^2^, in which about 60% and 40% were consumed by local inhabitants and inhabitants in other provinces, respectively. Figure 3 pictured the inter-provincial land use transfers in China. Inner Mongolia was the largest supplier of lands, which provided 442.8 thousand km^2^ land uses embodied in commodities to other provinces. Land uses in Inner Mongolia mainly flowed into Jiangsu (31.0 thousand km^2^), Shandong (30.1 thousand km^2^), Shanxi (28.2 thousand km^2^), Zhejiang (28.2 thousand km^2^), and Henan (24.7 thousand km^2^). Xinjiang, Tibet, Heilongjiang, and Gansu were other large land use exporters, which exported 310.3, 212.5, 184.6, and 114.1 thousand km^2^ land uses embedded in commodities, respectively. The provinces that received the most land uses from other provinces were Shandong, Guangdong, Zhejiang, Jiangsu, and Henan, which imported between 136.8 and 243.4 thousand km^2^ land uses. All the provinces were tightly connected with each other by inter-provincial trades of embodied land uses, forming an intensively interacted land use trade network (Figure 3). The bilateral links with the largest land use trades were Xinjiang–Shandong, Inner Mongolia–Jiangsu, Tibet–Shandong, Inner Mongolia–Shandong and Inner Mongolia–Shanxi, each of which involved over 28.2 thousand km^2^ land use transfers. The amounts of land use transfers in bilateral links presented a great variability, with 20% of the links representing over 55% of the total land use trades in China.

From the viewpoint of the net land use trade, one province is deemed a net land use exporter if it exports more land uses than it imports; by contrast, a net land use importer is a province that imports more land uses than it exports. Thirteen provinces were net land use exporters, based on inter-provincial land use trade accounting in China. This number was less than the number of provinces with a larger production-based land use than a consumption-based land use in the above analysis, because a part of production-based land use was exported abroad, which was not considered in the net land use trade accounting. The top five provinces net exporting the largest land uses embodied in products and services were Inner Mongolia, Xinjiang, Tibet, Heilongjiang, and Qinghai, with net land use outflows of 361.1, 274.3, 208.6, 103.4, and 80.6 thousand km^2^, respectively. Most of these net land exporters were located in the western China. In the other 18 provinces which net imported land uses via inter-provincial trades, Shandong ranked the first with the net land use inflow of 209.6 thousand km^2^, followed by Zhejiang (153.8 thousand km^2^), Guangdong (145.2 thousand km^2^), Jiangsu (125.0 thousand km^2^), and Henan (93.6 thousand km^2^). Most of these net land importers were located in the eastern coastal and central regions in China.

The inter-provincial trade has redistributed the land resources, leading to a reduction of differences in per capita consumption-based land uses in comparison to production-based land uses (Section 3.2). Overall, the provinces faced with high land supplying pressures have outsourced parts of their land uses to other provinces by net importing land uses. More equally distributed per capita consumption-based land uses than production-based land uses benefited a mitigation of land pressures in a number of provinces lack of land resources.

**Figure 4:**
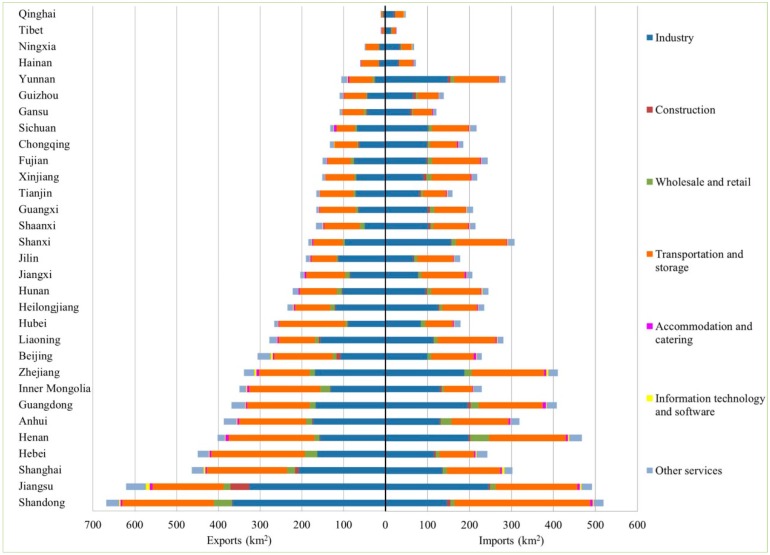
Land use exports and imports embedded in inter-provincial trades at sectoral level (excluding agricultural land uses). The provinces are ranked according to the total area of exported land uses.

Similar to production- and consumption-based land uses, land use transfers were dominated by agricultural land uses. Agricultural land uses represented between 47.13% and 99.99% of the total land use exports, and between 99.32% and 99.79% of the total land use imports in different provinces. All the ten provinces in Northwest China and Southwest China, which were net land use exporters, exported more agricultural lands than those they imported. Therefore, western provinces were the major agricultural land use exporters in inter-provincial trades. Figure 4 showed the land use exports and imports through inter-provincial trades with sectoral information (excluding agricultural land uses). Generally, western provinces exported less industrial and tertiary land uses than they imported, whereas central and eastern provinces were industrial and tertiary land net exporters. This pattern was tightly linked with the higher industrialization and socio-economic development levels in the eastern and central parts than the western part of China. In non-agricultural land uses, industry and transportation and storage sectors constituted high percentages of exported and imported land uses, which were consistent with their high shares of the total production- and consumption-based land uses.

### 3.4. Influencing Factors for Land Uses Transfers

**Figure 5:**
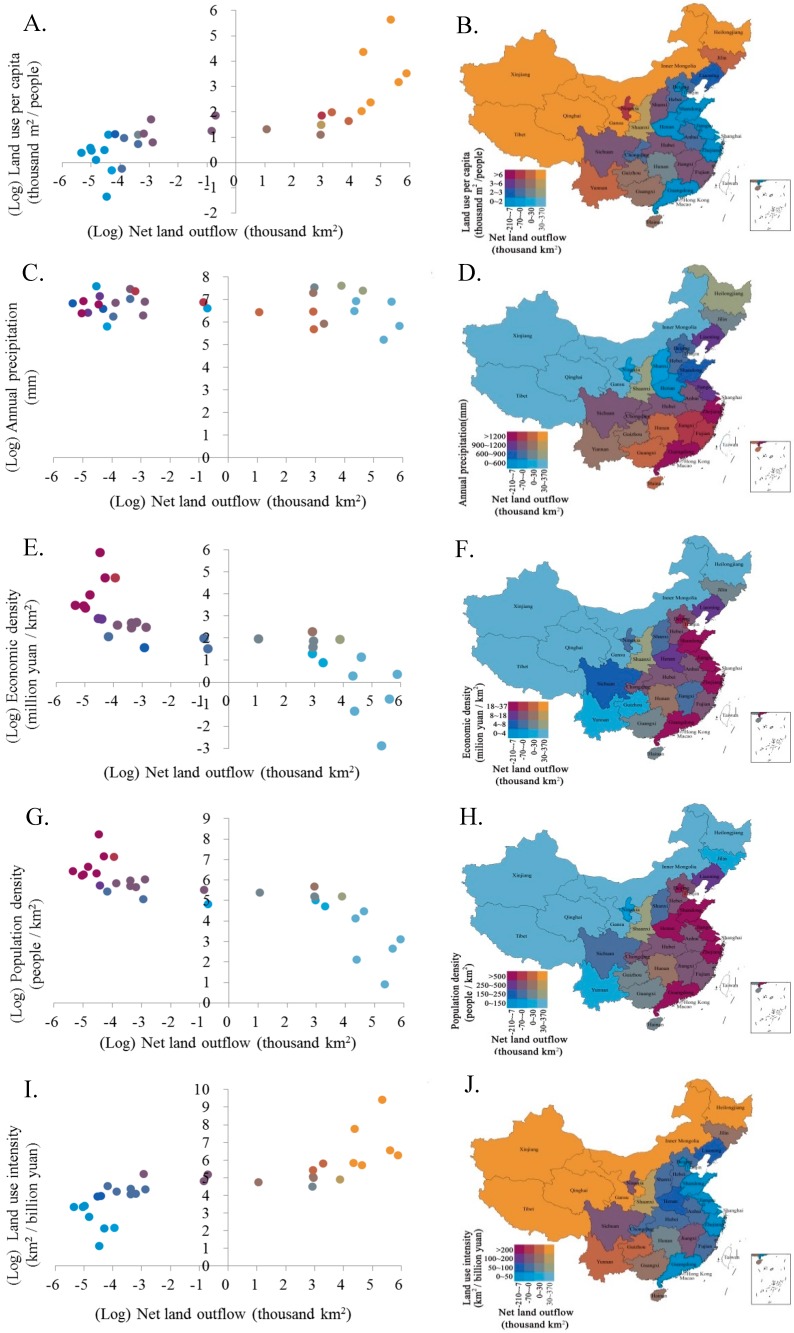
Bivariate maps between the net land use export and possible influencing factors: (**A**,**B**) Land uses per capita, (**C**,**D**) annual precipitation, (**E**,**F**) economic density, (**G**,**H**) population density, and (**I**,**J**) land use intensity.

Figure 5A,B showed the bivariate map of the net land use trade (net land use export was represented by a positive value) and land use per capita at the provincial level in China. Eastern coastal provinces (i.e., Shanghai, Beijing, Tianjin, Jiangsu, and Shandong), with low per capita land uses between 0.73 and 1.48 thousand m^2^, were net land use importers, whereas most northern and western provinces with high per capita land uses above 1.49 thousand m^2^ were land use exporting regions. The rank correlation coefficient between per capita land use and net land use trade was 0.90. A significant positive relation (at the 5% significance level) suggested that the availability of the land resources per capita was likely a driving force for the net land use trade in China, which drove embedded land uses be transferred from regions with high availability of land resources to regions with low availability. This was favored from a land resource management perspective, because the redistribution of land resources by net land use transfers was beneficial to land pressure mitigation in a number of provinces with low land availability.

Figure 5C,D presented the bivariate map of the net land use trade and annual precipitation in different provinces in China. Controlled by the Pacific and India Ocean monsoons in most regions, annual precipitation decreased gradually from the southeast coastal areas to northeast inlands in China. The rank correlation coefficient between the provincial annual precipitation and net land use trade was −0.4. A significant negative correlation was detected, suggesting that embodied land uses in commodities had a tendency to be transferred from relatively dry regions to wet regions by inter-provincial trades in China. This can possibly be explained by the fact that climatic variables usually had a significant correlation with land productivities. The generally low productivity of the lands in dry regions led to net land use export to wet regions with high land productivity, because the production of equivalent products in dry regions usually required more lands than in wet regions. However, the correlation between precipitation and net land use trade was the least in all the investigated correlations, as the absolute value of the rank correlation coefficient was the lowest.

Figure 5E,F illustrated the bivariate map of the net land use trade and economic density in different provinces in China. The economic density presented a decreasing pattern from eastern coastal provinces to west regions. Shanghai had the largest economic density, whereas Tibet was the province with the least economic density, which was only 1.7% of the economic density in Shanghai. The rank correlation coefficient between the economic density and net land use trade was −0.91. A significant negative correlation implied that the embodied land uses flowed from less developed regions with low economic density to more developed regions with high economic density to meet the high living standards of inhabitants there. This result was consistent to previous findings from global land use studies [24,30].

The bivariate map of the net land use trade and population density and their correlation were presented in Figure 5G,H. As the population density was highly correlated to economic density, the bivariate map resembled that of the net land use trade and economic density. The largest population density occurred in Shanghai, with 3754 persons per km^2^, whereas the least population density was in Tibet, with the population density only 5% that of Shanghai. Similarly, the population density had a significant negative effect on the net land use trade, with a rank correlation coefficient of −0.90, suggesting that land uses tended to be traded from less densely populated western regions to more densely populated eastern regions in China. The high demand of land uses in intensively populated east China was met by outsourcing land uses to less populated west part of China.

Figure 5I,J showed the spatial distribution of the net land use trade and land use intensity in provincial China. As lands were generally used and managed more efficiently in more developed regions, the land use intensity was highly correlated to economic intensity. Therefore, the land use trade and land use intensity presented a positive correlation with the rank correlation coefficient 0.90, indicating that regions with lower land use intensities tended to provide embodied land uses to regions with higher land use intensities through trades. This tendency led to loss of land uses in comparison to the case when all the products and services were produced locally. From the land use efficiency management perspective, this is not favored.

Overall, these bivariate map and correlation analyses suggested that in China, land resources embodied in inter-provincial trade inclined to flow from the provinces with less developed economy, abundant land resource, sparse population and low land use efficiency to provinces with more developed economy, scarce land resource, dense population and high land use efficiency. To a certain extent, this land use trade pattern can alleviate the land supplying pressure in rapidly urbanized and developed affluent regions. Meanwhile, for less developed regions, this pattern can raise local land use rate, create job opportunities and accelerate infrastructure construction [38]. In principle, land use transfers can promote land use reallocation to achieve more equality of land uses and higher land use efficiency at the national level. While the consumption-based land use was overall more equal than the production-based land use, inter-provincial land use transfers resulted in land losses from an efficiency perspective. Land grabbing may exert negative effects in deprived areas, including land quality deterioration, environment disruption, and biodiversity decrease [39].

## 4. Conclusions and Policy Implications

From a per capita perspective, China is poorly endowed with land resources, with much lower availability than the world average level. Due to rapid population growth and urbanization process, demands in lands for meeting the needs of food production and urbanization were increasing and the supply of the resource was faced with extreme pressures. Given the mismatch of land resources availability and population in China, land use transfers by inter-provincial trades of agricultural and other products may serve as an efficient measure to reallocate land resources between different provinces. In this study, the full view of China’s sectoral land uses at the provincial level was provided. The MRIO model was applied to conduct a systematic analysis of land uses in China, which included upstream resource consumption in the production chains and appropriates land uses to final consumers. In addition, natural and socioeconomic factors that are associated with the net land use transfers by inter-provincial trades were examined. Based on the results of this study, the following conclusions were drawn and corresponding policy implications were provided:

(1) The land use intensities presented large heterogeneity in varying industrial sectors and different provinces. The land use efficiencies in affluent provinces were generally higher than poor provinces. Agriculture was the most direct land use intensive sector. Differences in land use intensities in different provinces indicated high potential of efficiency improvement in a number of provinces. Sustainable agricultural management, including farmland circulation and nutrient recycling, should be encouraged, in order to increase the land productivity and reduce adverse environmental impacts. High efficiency agricultural technologies should be shared between provinces. Given that agricultural production in western China is dominated by decentralized household-based, small-scale crop and animal productions are encouraged to be managed on larger scales, to gain scale effect for more efficient uses of agricultural infrastructure and other resources. Industrial and tertiary sectors are also in the core of land use efficiency improvement, because they are generally located in urban areas where the land resources are precious due to limited availability. Circular economy, cleaner production and eco-design should be promoted to improve the overall resource efficiency.
(2) Agricultural land uses represented a major proportion of the production- and consumption-based land uses as well as inter-provincial land use transfers. For most provinces, agricultural land uses constituted over 99% of the total land uses. However, per capita agricultural land of China was only one third of the world average in 2012 [40]. Overall, agricultural land uses were transferred from western China to eastern China, whereas land uses embodied in non-agricultural products flowed from eastern China to western China. With the projection of the increasing population and rapid urbanization in the future, the conflicts between urban expansion and agricultural land conservation will become increasingly prominent. In this context, arable land protection is essential for assuring food security in China. Measures including strictly following the “red line” restriction of cultivated land and delimiting permanent basic farmland protective zone are necessary for this need. Economic instruments can be applied to prevent the value of precious land resources embedded in agricultural products being underestimated.
(3) A highly-interacting network of land use transfers between different provinces has been formed in China. Products and services involved in inter-provincial trades in China contained 2.3 million km^2^ land uses, which constituted approximately 40% of the total national land uses that were finally consumed in China. At the provincial level, main suppliers of lands were Inner Mongolia, Xinjiang, Tibet, Heilongjiang, and Gansu, whereas Shandong, Guangdong, Zhejiang, Jiangsu, and Henan received the most land uses embodied in trades. Per capita production-based land uses presented a larger variability than consumption-based land uses in China, implying that inter-provincial trades have reduced inequlaity of per capita land uses in China. Land resource scarce provinces with low per capita land availability have outsouced parts of their land uses by net importing lands from other provinces. Therefore, outsourcing land uses can be considered as a potential solution for mitigating land resource shortages in resource scarce regions. However, the dependence of food security in land use importing regions on other regions should not be neglected.
(4) The net land use transfers embodied in inter-provincial trades in China were closely related to socio-economic conditions, including the land use per capita, economic density, population density, and land use intensity. The impact of annual precipitation on net land outflow was relatively minor. Land uses embodied in trades inclined to flow from regions with less developed economy, sparse population, high land availability, and low land use efficiency to regions with advanced economy, dense population, low land availability, and high land use efficiency. This land grabbing may lead to land quality deterioration, environment disruption, and biodiversity decrease in deprived land exporting areas. Because deprived provinces are usually less capable of addressing these adverse impacts due to a lack of financial and technological means, an ecological compensation system needs to be established to provide these provinces with essential financial and technological aids to better cope with associated problems caused by land grabbing.

## Figures and Tables

**Table 1:** Summarized statistics and data sources of selected possible influencing factors.

Variable Dimension	Possible Influencing Factor	Unit	Range	Mean/Standard Deviation	Data Sources
Natural endowment	land use per capita	thousand m^2^/person	[0.26, 283.73]	16.69/50.92	[32,34]
	annual precipitation	mm	[188, 2028]	966/515	[31]
Socio-economic conditions	economic density	million yuan/km^2^	[0.06, 360.8]	29.2/66.4	[34]
	population density	People/km^2^	[2.5, 3754.0]	439.4/679.9	[34]
	land use intensity	km^2^/billion yuan	[3.1, 12,465.7]	615.9/2205.2	[32,34,35]

**Table 2:** Direct and total land use intensities of agricultural, industrial, and tertiary sectors in different provinces in China (unit: km^2^/billion yuan).

Province	Agriculture		Industry		Tertiary	
	Direct Land Use Intensity	Total Land Use Intensity	Direct Land Use Intensity	Total Land Use Intensity	Direct Land Use Intensity	Total Land Use Intensity
Beijing	283.5	410.3	0.13	3896.6	0.14	16.5
Tianjin	184.9	244.2	0.07	3229.1	0.17	19.3
Hebei	245.0	311.0	0.08	4264.5	0.39	17.4
Shanxi	773.3	938.9	0.11	4303.1	0.26	22.6
Inner Mongolia	3636.2	4271.4	0.12	30,637.8	0.34	125.1
Liaoning	280.9	380.9	0.10	6092.7	0.25	27.7
Jilin	659.5	829.0	0.13	9539.8	0.33	50.3
Heilongjiang	984.8	1152.6	0.19	12,570.2	0.46	44.1
Shanghai	105.9	169.8	0.20	2997.0	0.21	13.9
Jiangsu	114.7	148.4	0.08	3654.9	0.21	12.8
Zhejiang	325.1	369.5	0.10	4890.6	0.22	23.6
Anhui	299.7	376.5	0.14	5494.7	0.41	31.4
Fujian	359.3	419.2	0.09	4839.5	0.20	25.8
Jiangxi	596.0	688.4	0.12	6729.2	0.41	30.5
Shandong	145.3	246.0	0.09	6825.1	0.29	20.1
Henan	186.9	274.1	0.08	5818.0	0.43	20.3
Hubei	321.4	393.5	0.16	6045.2	0.32	20.4
Hunan	368.0	449.2	0.10	5652.7	0.26	22.0
Guangdong	320.6	381.1	0.09	2718.8	0.21	16.9
Guangxi	536.0	639.5	0.13	7121.4	0.40	26.6
Hainan	267.8	330.5	0.11	4807.3	0.25	21.5
Chongqing	499.3	632.9	0.11	5753.0	0.28	22.3
Sichuan	778.4	940.2	0.12	11,090.7	0.32	52.3
Guizhou	1044.3	1266.6	0.16	6863.6	0.33	33.1
Yunnan	1206.7	1487.5	0.07	8329.1	0.31	46.4
Tibet	71,087.4	87,495.9	0.16	97,939.0	0.50	1239.2
Shaanxi	805.2	995.0	0.06	5996.5	0.29	33.8
Gansu	1594.2	1880.7	0.13	9342.3	0.41	52.4
Qinghai	16,831.9	18,697.1	0.08	41,623.7	0.31	189.6
Ningxia	1034.9	1199.6	0.09	6356.2	0.43	33.5
Xinjiang	2521.8	3061.7	0.22	12,112.2	0.61	94.2
